# Differences in DNA curvature-related sequence periodicity between prokaryotic chromosomes and phages, and relationship to chromosomal prophage content

**DOI:** 10.1186/1471-2164-13-188

**Published:** 2012-05-15

**Authors:** Jacob Abel, Jan Mrázek

**Affiliations:** 1 Department of Microbiology, University of Georgia, Athens, GA, 30602, USA; 2 Institute of Bioinformatics, University of Georgia, Athens, GA, 30602, USA; 3 Present address: School of Medicine, University of Pittsburgh, Pittsburgh, PA, 15261, USA

**Keywords:** Prokaryotic genome, DNA bending, Nucleoid structure, Prophage integration, Virus, DNA packaging

## Abstract

**Background:**

Periodic spacing of A-tracts (short runs of A or T) with the DNA helical period of ~10–11 bp is characteristic of intrinsically bent DNA. In eukaryotes, the DNA bending is related to chromatin structure and nucleosome positioning. However, the physiological role of strong sequence periodicity detected in many prokaryotic genomes is not clear.

**Results:**

We developed measures of intensity and persistency of DNA curvature-related sequence periodicity and applied them to prokaryotic chromosomes and phages. The results indicate that strong periodic signals present in chromosomes are generally absent in phage genomes. Moreover, chromosomes containing prophages are less likely to possess a persistent periodic signal than chromosomes with no prophages.

**Conclusions:**

Absence of DNA curvature-related sequence periodicity in phages could arise from constraints associated with DNA packaging in the viral capsid. Lack of prophages in chromosomes with persistent periodic signal suggests that the sequence periodicity and concomitant DNA curvature could play a role in protecting the chromosomes from integration of phage DNA.

## Background

Periodic spacing of A-tracts (short runs of A or T) with the DNA helical period of ~10–11 bp is associated with intrinsic DNA curvature [1,2]. In eukaryotes, this periodicity is a major component of the nucleosome positioning signal [1,3-7]. The DNA sequence periodicity can influence properties of the chromatin and expression of the encoded genes. For example, the “hyperperiodic regions” of the *Caenorhabditis elegans* genome are subject to particular types of histone modifications and contain mostly germ-line specific genes [8-10].

Strong periodic patterns with the characteristic period of ~10–11 bp were also detected in many prokaryotic genomes [9,11-16]. The biological role of the sequence periodicity and DNA curvature in prokaryotes is not clear, although several possible roles or causes of the 10–11 bp periodicity have been proposed. Zhurkin pointed out that a similar periodic signal can arise from amphipathic α-helices in proteins [17]. α-helices feature a helical period of about 3.6 residues per turn, which in DNA translates to an ~10.8 bp period. However, presence of the periodic patterns in both protein-coding and noncoding regions, as well as the extent of the periodicity beyond distances typical of α-helix lengths suggest that the sequence periodicity related to α-helices in proteins is not the only cause of the observed strong periodic signal in prokaryotic DNA [11,12]. DNA bending affects DNA-protein interactions and curved DNA segments are often associated with promoters, where they can influence interactions between DNA and transcription factors [18,19]. The sequence periodicity could also promote a particular mode of supercoiling: Herzel and coworkers proposed that ~11 bp periodicity found in most bacteria can relate to predominant negative supercoiling whereas ~10 bp periodicity of some archaeal genomes could promote positive supercoiling [9,13]. Tolstorukov and coworkers analyzed clusters of periodically spaced. A-tracts in *E. coli* and other bacterial genomes, and proffered a hypothesis that the sequence periodicity-driven intrinsically curved segments contribute to the formation and stability of supercoiled DNA loops that constitute bacterial nucleoid [14]. It is possible that the DNA sequence periodicity in prokaryotes can play multiple roles, including facilitating transcription initiation, contributing to DNA folding in the nucleoid, and promoting positive or negative supercoiling, but most likely to different extent in different genomes.

Whereas earlier works focused on measuring the predominant period in a whole genome, we recently developed a technique that allows comparing the intensity of the periodic signal among different genomes or different regions of the same genome [20]. Comparisons among more than 1000 prokaryotic chromosomes revealed major differences in the intensity of the periodic signal among different genomes as well as varying levels of intrachromosomal heterogeneity [11]. In most prokaryotic chromosomes, a strong DNA curvature-related periodicity is restricted to short chromosomal segments. By contrast, some genomes feature a persistently strong periodic signal covering majority of the chromosome length. Finally, some genomes exhibit hardly any periodic signal at all. We attributed the differences to presumed organism-specific differences in nucleoid structure and also noted that intrachromosomal heterogeneity could be related to differences in gene expression in different sections of the chromosome [11]. We now extended the analysis of DNA curvature-related sequence periodicity in prokaryotes to large bacteriophages. Comparisons with chromosomal DNA indicate that phages generally lack a strong periodic signal. Moreover, chromosomes that contain prophages tend to have weaker or less persistent sequence periodicity, suggesting that the periodicity and concomitant DNA curvature could also play a role in protecting the chromosome from phage integration.

## Results and discussion

### Strong periodic signals found in prokaryotic chromosomes are absent in bacteriophages

**Figure 1:**
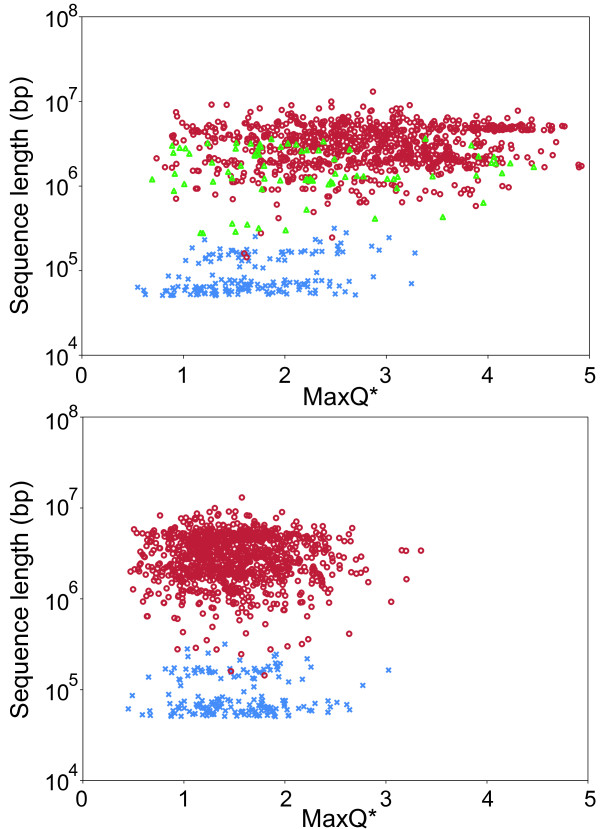
**Intensity of DNA curvature-related periodic signal in prokaryotic chromosomes and bacteriophages and comparison with random sequences.** Top: The MaxQ* index (see Methods) is used as a measure of the intensity of DNA curvature-related periodicity in the analyzed sequence. Each chromosome or phage is represented by a point with the horizontal coordinate determined by the MaxQ* value and the vertical coordinate by the sequence length in bp. Primary chromosomes (the largest chromosome of a genome) are shown as red circles, secondary chromosomes as green triangles, and phages as blue crosses. Bottom: An analogous plot for randomized sequences. A random sequence was generated for each chromosome (red circles) and phage (blue crosses) reproducing the length and G + C content of the original sequence. The random sequences were subsequently processed in the same way as the original sequences in the top panel. The Genome Randomizer software was used to generate the random sequences (http://www.cmbl.uga.edu/downloads/programs/Genome_Randomizer/, [21]).

The distribution of the intensity of DNA curvature-related periodic signal in sequences of 1025 prokaryotic chromosomes and 168 large phages (with genomes larger than 50 kb) is shown in Figure 1, top panel. The signal intensity is measured by the MaxQ* index (see Methods). There is a clear distinction between chromosomes and phages in terms of sequence periodicity, with phages exhibiting lower MaxQ* values. Comparisons among random sequences show that the differences between chromosomes and phages are not a simple consequence of differences in sequence lengths, as both randomized phage and chromosome sequences occupy approximately the same range of MaxQ* (Figure 1, bottom panel). In a separate test, we compared the 160 phages in our dataset with genome sizes between 50 and 200 kb with a collection of randomly selected chromosomal segments with lengths in the same 50–200 kb range. One such segment was extracted from each of the 1025 chromosomes. The mean MaxQ* for phages was 1.67 compared to 1.89 in the random chromosomal segments of similar sizes. The difference is significant with p = 0.0003 by the Mann–Whitney *U*-test, confirming that the differences between the phages and chromosomes cannot be explained solely by different genome sizes.

Phage DNA undergoes processes that do not apply to chromosomal DNA, most importantly packaging in the capsid. While different viruses utilize different DNA packaging mechanisms, one common aspect is the spatial constraint of the capsid interior. To satisfy the spatial constraints, the DNA is wrapped in rather regular tight loops that minimize the bending stress on the DNA molecule [22,23]. Extensive intrinsic DNA bending could increase the stress from structural deformation imposed by tight DNA packaging and the constraints related to DNA packaging represent one possible scenario to explain the absence of strong sequence periodicity found in chromosomal DNA.

### Chromosomes with a persistent periodic signal are less likely to contain prophages

The observation that phage genomes lack the DNA curvature-related sequence periodicity led us to investigate whether there is any relationship between the sequence periodicity and presence or absence of prophages in a chromosome. Of 665 chromosomal sequences surveyed by prophinder [24], 358 (53.83%) were predicted to contain at least one integrated prophage, while the remaining 307 contained no predicted prophages. Some prokaryotic chromosomes contain exceptionally persistent periodic signals throughout the chromosome [11], here defined as having a MaxMax score ≥ 20. 23 of 307 (7.49%) chromosomes with no prophages possessed exceptionally persistent periodic signals compared with 5 out of 358 (1.40%) sequences with at least one integrated prophage. This difference is statistically significant (p < 10^−4^ by Fisher’s exact test) and indicates an increased likelihood that a genome with persistent periodic signal does not contain a prophage.

The significant relationship between the sequence periodicity and presence or absence of a prophage in a chromosome was further confirmed by comparisons of the values of Max2, Max3, and MaxMax indices between the chromosomes with and without prophages using the Mann–Whitney *U* test (Table 1). The Max2, Max3, and MaxMax indices represent different ways to measure the persistency of a dominant periodic signal throughout a chromosome (see Methods). To reduce a possible bias from inclusion of multiple strains of the same or closely related species we selected randomly only one strain of each species for the statistical test, and repeated the test 1000 times. For Max3 and MaxMax indices, all 1000 tests confirmed a significant difference between prophage-containing and prophage-lacking chromosomes (using the standard criterion p < 5%), whereas 208 of the 1000 tests yielded a significant difference in Max2 values (Table 1).

**Table 1:** **Results of Mann–Whitney U tests for significant differences in periodicity indices between sequences with and without prophages**^**a**^

**Index**	**Number of tests that yielded p ≤ 5%**
Max2	208
Max3	1000
MaxMax	1000

^a^ We used a technique similar to the bootstrap test to reduce the data bias towards model organisms, which often have multiple sequenced strains of the same species. A single genome was randomly selected to represent each species in our dataset while all other genomes of the same species (if more than one genome was available) were excluded from the subsequent analysis. The resulting dataset was divided into two groups based on whether the chromosome contained a prophage or not. The Max2, Max3, and MaxMax indices in both groups were subsequently compared using the Mann–Whitney test. The test was repeated 1000 times with different random selections of representative genomes. The table shows the number of tests that led to rejecting the null hypothesis when using a standard 5% probability cutoff.

**Figure 2:**
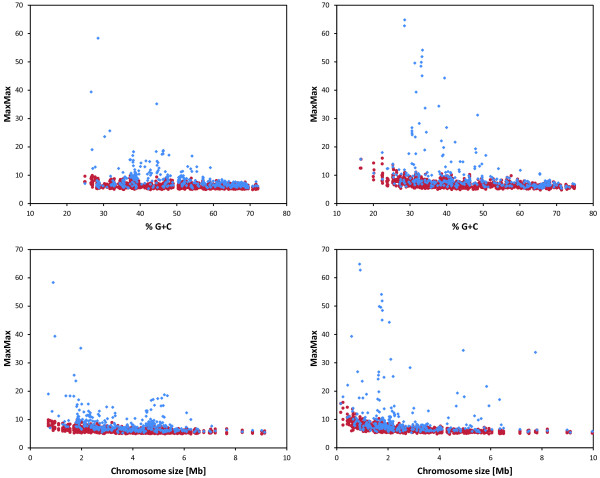
**Persistency of the periodic signal in chromosomes with and without prophages.** Top left: The MaxMax value (a measure of persistency of a dominant sequence periodicity in a chromosome) is plotted for each chromosome with at least one prophage against the G + C content (blue diamonds). Analogous data for randomized sequences preserving the overall G + C content and size of the original sequence are shown for comparison (red circles). Bottom left: A similar plot but showing the MaxMax values as a function of the chromosome size. Top right and bottom right: the same plots for chromosomes with no prophages. The classification of chromosomes with and without prophages relies on Prophinder predictions.

A visual demonstration of the difference between chromosomes with and without prophages in terms of persistency of the sequence periodicity is provided in Figure 2. Chromosomes with high MaxMax values generally do not contain prophages. The plots in Figure 2 also show that the chromosomes with one or more prophage tend to be larger than those with no prophages, and that no prophages were detected in genomes with extremely low GC content (< ~25%).

These results lead us to speculate that the sequence periodicity and DNA curvature in prokaryotic chromosomes could play a role in protecting the genomic DNA from phage integration. Tolstorukov and coworkers [14] hypothesized that curved DNA segments in bacterial chromosomes promote nucleoid compaction and increase the stability of the DNA complexes with architectural proteins. Our subsequent analysis of sequence periodicity in prokaryotic chromosomes showed that most prokaryotic chromosomes contain alternating segments of strong and weak periodicity. We hypothesized that the heterogeneity of sequence periodicity could reflect structural heterogeneity of the nucleoid, where more structurally stable (rigid) sections characterized by strong sequence periodicity alternate with less stable (flexible) segments characterized by weak periodicity [11]. It is possible that rigid sections of the nucleoid are less susceptible to phage integration, and chromosomes with persistent sequence periodicity are (partially) protected from phage integration. This notion is also consistent with our earlier finding that highly expressed genes tend to be located in non-periodic (structurally flexible) segments of the chromosome [11], as both transcription and phage integration likely require local unfolding of the nucleoid structure.

Integration of multiple prophages could also in some cases decrease the persistency of the periodic signal in a chromosome because, as we demonstrated above, phages tend to lack the sequence periodicity. Integrated prophages can comprise up to 10–20% of the chromosome size [25], and additions of such an amount of non-periodic DNA into a chromosome with otherwise persistent sequence periodicity could decrease the values of the Max2, Max3, and MaxMax indices. However, this mechanism applies only to chromosomes with very high prophage content as integration of only a few prophages would have little effect on our measures of the persistency of the periodic signal.

### Intragenomic heterogeneity of sequence periodicity may affect the prophage integration sites

**Figure 3:**
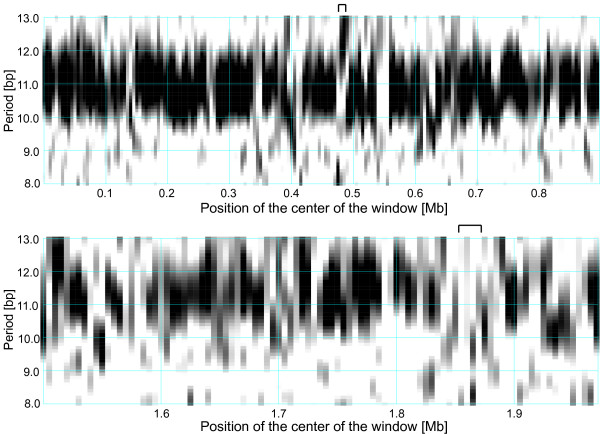
**Absence of DNA curvature-related periodicity at prophage integration sites.** The top panel shows a periodicity scan of the *Mycoplasma hyopneumoniae* J chromosome and the bottom panel shows a periodicity scan of a section of the *Campylobacter curvus* 525.92 chromosome containing a prophage. The intensity of the periodic signal for a given chromosomal location shown on the horizontal axis and the period shown on the vertical axis is signified by the level of grey. White regions correspond to a weak periodic signal while black indicates a strong periodicity (See Methods for details). A 10 kb window was moved in steps of 5 kb. Locations of prophages predicted by the Prophinder are marked by the bars above the plot. *M. hyopneumoniae* has a predicted prophage at positions 469,140–481,698 bp and *C. curvus* at 1,857,093–1,873,803 bp.

There are a few chromosomal sequences that do not fit the general pattern described above and contain a predicted prophage despite exhibiting a relatively persistent A-tract periodicity. We investigated the interplay of sequence periodicity and phage integration in the genomes of *Mycoplasma hyopneumoniae* J (MaxMax = 58%) and *Campylobacter curvus* 525.92 (MaxMax = 35%), which contain one prophage each according to the Prophinder search (Figure 3).

*M. hyopneumoniae* J possesses a remarkably strong and persistent periodic signal with the dominant period 10.9 bp, which spans almost the entire genome and features only few non-periodic regions [11]. One such region (~470 kb – 485 kb) features a signal with a period 12–13 bp, but lacks the periodicity typically associated with DNA curvature (~10–11 bp). In particular, this region lacks the ~10.9 bp periodicity, which is characteristic of the bulk of the chromosome (Figure 3). Furthermore, this region coincides with the insertion site of a predicted prophage at position 469,140–481,698 bp. The *C. curvus* 525.92 chromosome also possesses a strong and relatively persistent periodic signal with a predominant period ~11.4 bp. A predicted prophage resides at position 1,857,093–1,873,803 bp, which is located in a segment that lacks the periodicity characteristic of the bulk of the chromosome (Figure 3).

In the previous work, we proffered that the bacterial nucleoid consists of a mosaic of DNA loops with strong sequence periodicity indicative of a more rigid conformation and regions of weaker periodicity, which likely confers a more flexible loop configuration [11]. We have also shown that highly expressed genes tend to be localized in regions of weak periodicity. We hypothesize that phages may prefer integrating in structurally more flexible regions of the chromosomes characterized by lower content of intrinsically bent DNA, a weak sequence periodicity, and probably higher transcriptional activity.

## Conclusions

Several possible roles were proposed for DNA curvature and concomitant sequence periodicity in prokaryotes but its exact biological function is not well understood. Our comparisons of the A-tract periodicity between prokaryotic chromosomes and phages suggests that the DNA curvature could help protect the chromosomes from phage integration, most likely as a consequence of its contribution to nucleoid compaction.

## Methods

### DNA sequences

Complete DNA sequences of 1025 prokaryotic chromosomes were downloaded from the NCBI FTP server (ftp://ftp.ncbi.nih.gov/genomes/Bacteria/). 168 complete bacteriophage genomes of size ≥50 kb were retrieved from the NCBI Entrez database.

### Assessing sequence periodicity in the whole genome (periodicity plot)

We devised the Periodicity Plot (PerPlot) technique to assess the DNA curvature-related periodicity in the whole genome context [11,20]. The PerPlot algorithm starts by constructing a histogram N(s) of spacings between pairs of “A-tracts”, which can be defined as various combinations of A and T nucleotides. In this work, we use the A-tracts defined as dinucleotides AA and TT (the “A2T2” method [11]). The function N(s) refers to the number of times N a pair of A-tracts occurs at the mutual distance s anywhere in the genome; all partially overlapping A-tracts are included in the count. The histogram N(s) is subsequently processed in a series of steps to reduce noise and various artifacts. First, it is normalized relative to expected counts based on random distribution of A-tracts. The 3-bp periodic signal arising from biased codon usage in genes is removed with a 3-bp sliding window average and a slope in the plot that can arise from varying nucleotide composition along the sequence is eliminated by subtracting a parabolic regression from the observed values [11]. A section of the modified histogram between values s_min_ and s_max_ is converted to a power spectrum by Fourier transform. We use the s_min_ and s_max_ values 30 and 100 bp, respectively. This is the range of distances where the DNA curvature-related periodicity is expected to be most pronounced in most genomes [11,12]. To facilitate comparisons among different genomes we scale the power spectrum such that the average value between periods 5 and 20 bp equals 1. We refer to this normalized power spectrum as Q*(P) See references for detailed description of the methodology [11,20].

The normalized power spectrum allows extracting two indices that characterize the dominant periodic signal: MaxQ is the height of the highest peak in the spectrum and measures to what extent the dominant peak exceeds the noise in the range of periods 5–20 bp. PMaxQ is the position of the highest peak and measures the dominant period in the A-tract spacing. Simulations with randomized genomes showed that approximately 1.5% of random sequences yield MaxQ ≥ 3.0 [11,20]. To reduce the possibility that peaks unrelated to DNA curvature (e.g., with a period distant from the DNA helical period of ~10.5 bp, which can sometimes arise from sequence repeats in an absence of a strong DNA curvature-related signal) are misinterpreted as DNA curvature-related peaks we use modified indices MaxQ* and PMaxQ* in this work. The MaxQ* and PMaxQ* indices refer to the maximum value of the scaled power spectrum Q^*^(P) within the range of periods 9.5–11.5 bp (the 5–20 bp range is still used in the normalization used to convert the raw power spectrum Q(P) into Q*(P)).

### Assessing intrachromosomal heterogeneity of the periodic signal (periodicity scan)

An important question related to the interpretation of the periodic signal detected in a genome is whether the periodicity is evenly distributed throughout the genome or concentrated in a few periodic regions. The Periodicity Scan (PerScan) approach applies the PerPlot technique in a sliding window mode: a sliding window, typically 10 kb length, is moved along the genomic DNA sequence and the scaled power spectrum Q*(P) is constructed for each window position. We display the results as grayscale heat map plots where the horizontal axis shows the position of the sliding window, the vertical axis determines the period, and the intensity of the periodic signal Q*(P) for the given period and window position is signified by the level of grey in the plot area (black indicates a strong periodicity while white refers to absence of a significant periodic signal). The PerScan technique allows measuring the persistency of the dominant periodic signal as a fraction of the genome (percentage of the sliding window positions) that shows a strong periodic signal with approximately the same period. For this purpose, we define three pairs of indices: PMax3 refers to the period for which the largest fraction of the window positions has the normalized power spectrum value Q*(PMax3) ≥ 3.0 and Max3 is that fraction of windows (measured in %). PMax2 and Max2 are defined analogously using the criterion Q*(PMax2) ≥ 2.0. PMaxMax is the period for which the largest number of windows yields PMax in the range PMaxMax ± 0.2 and MaxMax is the corresponding percentage of the sliding window positions. The Max2, Max3, and MaxMax indices offer alternative quantitative measures of the persistency of the dominant periodic signal throughout the genome. For more details, see references [11,20] or the description of the PerPlot and PerScan software online (http://www.cmbl.uga.edu/software.html).

### Statistical assessments

Differences in average MaxQ* and differences in Max2, Max3, and MaxMax scores between sequences with and without integrated prophages were evaluated by the Mann–Whitney *U* test implemented in the R statistical computing environment (http://www.R-project.org/).

### Prophage detection

Using the pre-processed bacterial genome listing in the ACLAME database (http://aclame.ulb.ac.be/Tools/Prophinder/) [24,26], chromosomes were separated into two groups, those with at least one integrated prophage detected by prophinder and those without predicted prophages. For this analysis, sequences that were absent either in our 1,025 prokaryotic chromosome dataset or in the ACLAME database were discarded. The authors of prophinder found its predictions to carry a sensitivity of 79% and a positive predictive value of 94%. The uncertainty in the predictions can lead to some genomes being misclassified in terms of presence or absence of prophages. However, a limited number of misclassified sequences should not affect our conclusions regarding general differences between chromosomes with and without integrated prophages.

## Competing interests

The authors declare that they have no competing interests.

## Authors’ contributions

JA performed the research and prepared the first draft of the manuscript. JM conceived of the study and wrote parts of the manuscript. All authors read and approved the final manuscript.
